# High score of LDH plus dNLR predicts poor survival in patients with HER2-positive advanced breast cancer treated with trastuzumab emtansine

**DOI:** 10.1186/s12885-021-09131-6

**Published:** 2022-01-03

**Authors:** Liru Li, Lin Ai, Lin Jia, Lei Zhang, Boya Lei, Qingyuan Zhang

**Affiliations:** Internal Medicine-Oncology, Harbin Medical University Cancer Hospital, Harbin, 150081 Heilongjiang China

**Keywords:** dNLR, LDH, T-DM1, HER2 positive, Prognosis

## Abstract

**Objective:**

To investigate the prognostic value of derived neutrophil to lymphocyte ratio (dNLR) and lactate dehydrogenase (LDH) in patients with advanced HER2 positive breast cancer treated with trastuzumab emtansine.

**Methods:**

Fifty one patients with advanced HER2 positive breast cancer who received T-DM1 treatment in Harbin Medical University Cancer Hospital were selected. The clinical data and blood test indexes were collected, and the ROC curve determined the optimal cut-off value. Kaplan-Meier survival curve and Cox regression model was used to analyze the effect of different levels of dNLR,LDH,LNI (dNLR combined with LDH index) before and after T-DM1 treatment on the survival of patients.

**Results:**

The median PFS and OS of the patients with advanced HER2 positive breast cancer who received T-DM1 treatment were 6.9 months and 22.2 months, respectively. The optimal cut-off value of LDH and dNLR before T-DM1 treatment was 244 U / L (*P* = 0.003) and 1.985 (*P* = 0.013), respectively. Higher LDH and dNLR were significantly correlated with shorter median PFS and OS (*P* < 0.05). The median PFS of patients with LNI (0), LNI (1) and LNI (2) were 8.1 months, 5.5 months and 2.3 months, respectively, *P* = 0.007. Univariate and multivariate analysis showed that LDH > 244 U / L, dNLR > 1.985, LNI > 0, ECOG ≥1 and HER-2 (IHC2 +, FISH+) before the T-DM1 treatment were the poor prognostic factors. LDH uptrend after the T-DM1 treatment also predicted poor prognosis.

**Conclusion:**

Serum LDH > 244 U / L and dNLR > 1.985 before the T-DM1 treatment were prognostic risk factors for patients with advanced HER2 positive breast cancer receiving T-DM1 treatment. The higher LNI score was significantly associated with shorter PFS and OS. LDH uptrend after T-DM1 treatment was also related to the poor prognosis.

## Background

Breast cancer is the most common cancer among women in the world. Approximately 20% of breast cancers over-express human epidermal growth factor receptor 2 (HER2). In the past, patients with HER2-positive breast cancer generally had unfavorable outcome compared with HER2 negative cancers, but the prognosis of HER2-positive locally advanced or metastatic breast cancers (MBCs) has dramatically improved due to the introduction of trastuzumab, pertuzumab, and trastuzumab emtansine (T-DM1). T-DM1 is an antibody-drug conjugate which combines trastuzumab and the cytotoxic drug DM1 via a nonreducible thioether linker and it has been recommended as the standard second-line therapy of advanced breast cancer [1], which was associated with an objective response of 43.6% (95% confidence interval [CI], 38.6 to 48.6) and a median duration of progression-free survival of 9.6 months when the drug was administered after trastuzumab and a taxane [2]. Despite most patients can be controlled with T-DM1, there are still some patients who do not respond to the treatment. Biomarkers that predict the treatment efficacy of T-DM1 remain unknown.

Several studies have shown that serum LDH levels are associated with the prognosis of various tumors. Dynamic monitoring of serum LDH level changes can predict the efficacy and prognosis of chemotherapy in patients with breast cancer [3]. Notably, a meta-analysis, studies conducted in patients with several cancer types, confirmed that high serum LDH levels were associated with shorter PFS and OS in various cancers [4]. It may be caused by several factors, such as LDH is involved in the anaerobic glycolysis process of tumor growth and proliferation [5], and LDH can enable cancer cells to escape immune response by inhibiting CD8 + T lymphocytes and natural killing (NK) activation [6]. In addition, LDH can also promote tumor angiogenesis, cell migration and metastasis by increasing the expression of vascular endothelial growth factor (VEGF) [7].

In addition, inflammatory indicators are also believed to be related to the prognosis of various tumors. Several studies have used derived neutrophil-to-lymphocyte ratio (dNLR) as a prognostic indicator, and increased dNLR value is associated with poor prognosis of various malignant tumors including urothelial carcinoma [8, 9], breast cancer [10] and lymphoma [11, 12]. dNLR can reflect the immune state of the tumor microenvironment. Lymphocytes are involved in inhibiting tumor cells’ proliferation and metastasis by regulating cytotoxic cell and cytokine production to enhance immunity [13], while neutrophils can inhibit lymphokine activated to exert anti-tumor immune function [14]. Unbalanced levels of neutrophils and lymphocytes can result in tumor metastasis and poor prognosis.

In tumor-bearing mice treated with T-DM1, survival was reduced by depleting antibodies which inhibit the function of CD4+ and CD8+ T cells. Based on these results, T-DM1-induced efficacy may be partly mediated through immunity [15]. The combined detection of serum LDH and dNLR is also used to judge the efficacy and prognosis of cancer immunotherapy, and it shows high specificity. However, its value in the efficacy and prognosis of patients with metastatic HER2 positive breast cancer who were treated with T-DM1 is still unknown. The present study aims to evaluate the prognostic significance of LDH and dNLR in patients with advanced HER2 positive breast cancer treated with T-DM1, and investigate whether LDH and dNLR was able to predict treatment response to T-DM1.

## Methods

### Patients selection

We retrospectively analyzed the data of HER-2 positive advanced breast cancer patients who received T-DM1 treatment from May 2016 to November 2018 in Harbin medical university cancer hospital. Patients with insufficient clinical data or who discontinued treatment after the first cycle were excluded from the study. Demographic, clinical, and pathological patients characteristics were retrieved from medical records. The study protocol was approved by the Institutional Ethics Committee of Harbin medical university cancer hospital. All patients signed an informed consent to allow the use of their data for research purposes. All clinical and follow-up data were collected in November 2020.

### Blood parameters and ratios

Complete blood count and LDH(U/L) level at baseline within 1 week before the first T-DM1 treatment were collected (baseline pre-treatment sample), and dNLR which was defined as neutrophils/(leucocytes-neutrophils) was calculated. We used LNI to identify patients at high-risk of progression or death. LNI was defined as the combination of dNLR greater than the threshold value and LDH greater than ULN, which separated patients in three different risk groups (Good: 0 factor; Intermediate: 1 factor; Poor: 2 factors). Complete blood count and LDH (U/L) level were also extracted after 3 weeks ± 1 week of the first T-DM1 treatment (post-treatment sample). We divided the changing trends of LDH into three groups (down, steady and up) according to comparing the changes of LDH after T-DM1 treatment with before treatment, and steady was defined as LDH changed between 20 U/L. Similarly, dNLR also had three changing trends, and dNLR changed between 0.2 was identified as steady.

### Observation indicators

The efficacy of the treatment was evaluated by computerized tomography (CT). The therapeutic effective rate was calculated using the Response Evaluation Criteria 1.1 in Solid Tumors (RECIST 1.1) as a reference and the patients were separated into four stages, based on complete response (CR), partial response (PR), stable disease (SD) and progressive disease (PD). PFS was calculated from T-DM1 treatment start to the date of radiological or clinical documentation of PD, last follow-up or death, whichever occurred first (censored at last follow-up for patients alive and without PD). OS was calculated from experimental treatment start to the date of death or last follow-up (censored at last follow-up for patients alive). The adverse event grade of thrombocytopenia after 1 week of the first T-DM1 treatment was judged by National Cancer Institute Common Terminology Criteria for Adverse Events (CTCAE) v5.0 .

### Statistical analysis

We used IBM SPSS program version 25.0 to perform the statistical analysis. The means and medians of the variables were calculated by descriptive analysis. The cut-off value of LDH and dNLR were calculated by ROC curve. Patient characteristics of different groups and efficacy recist (total responded, partial responded, stable, and progressed) were compared using × 2 test for quantitative data or a Fisher exact probability test for categorical data. Kaplan-Meier method was used for survival analysis and constructing survival curves. Baseline LDH, dNLR, LNI and the changing trends of LDH and dNLR were calculated and along with other characteristics, correlated with progression-free survival (PFS) and overall survival (OS) in univariate and multivariate analyses. Comparison between survival curves was completed using the log-rank test. Hazard ratios (HRs) together with 95% confidence intervals (CI) were provided. *P* value less than 0.05 was reckoned as significant for all the analyses.

## Results

Baseline patient characteristics are summarized in Table 1. A total of 51 patients with complete clinical data were included in the study. As of November 2020, 2 patients did not experience disease progression and 36 patients died. The median PFS was 6.9 months (range 4.5–9.3) and median OS was 22.2 months (range 12.7–31.7).

**Table 1:** Baseline characteristics of 51 HER2-positive advanced breast cancer patients With T-DM1 Treatment

Variable	Number(%)
**Age (years)**	
< 60	43(84.3)
≥ 60	8(15.7)
**ECOG PS**	
0	30(58.8)
1 and 2	21(41.2)
**Menstrual status**	
Menopause	39(76.5)
Non-menopause	12(23.5)
**CTCAE grades**	
≤ 2	38(74.5)
> 2	13(25.5)
**Treatment lines**	
**<** 2	32(62.7)
≥ 2	19(37.3)
**Previous treatment**	
Trastuzumab	27(52.9)
Trastuzumab plus pertuzumab	12(23.5)
Lapatinib / Pyrotinib	2(3.9)
Others	10(19.6)
**BMC status**	
De novo	5(9.8)
Recurrent	46(90.2)
**Metastatic sites**	
Brain	10(19.6)
Bone	25(49.0)
Lung	13(25.5)
Liver	27(52.9)
Chest wall	12(23.5)
Lymph node	22(43.1)
**HER2 status**	
HER2(IHC2+)FISH(+)	6(11.8)
HER2(IHC3+)	45(88.2)
**HR status**	
Positive	19(37.3)
Negative	32(62.7)
**Number of metastatic sites**	
≤ 2	9(17.6)
> 2	42(82.4)
**Prior surgery**	
Yes	46(90.2)
No	5(9.8)
**dNLR**	
> 1.985	15(29.4)
≤ 1.985	36 (70.6)
**LDH**	
> 244	21(41.2)
≤ 244	30(58.8)
**LNI scores**	
0	24(47.1)
1	17(33.3)
2	10(19.6)
**LDH changing trend**	
Down	16(31.4)
Steady	15(29.4)
Up	20(39.2)
**dNLR changing trend**	
Down	26(51.0)
Steady	13(25.5)
Up	12(23.5)

### LDH,dNLR and LNI

The optimal cut-off values that were determined by the ROC for the LDH and dNLR within 1 week before the first T-DM1 treatment are shown in Table 2. The optimal cut-off values for the LDH and dNLR were 244 U/L(*P* = 0.003) and 1.985(*P* = 0.013), respectively. The corresponding AUCs for the LDH and dNLR were 0.793 and 0.694, respectively.

**Table 2:** Receiver operating characteristics analyses of LDH and dNLR

Variables	AUC	Cut-off Value	Sensitivity	Specificity	***P***
LDH	0.793	**244**	0.621	0.812	**0.003**
dNLR	0.694	**1.985**	0.414	1.000	**0.013**

According to these cut-off values, the patients were then separated into two groups (low-value group vs. high-value group) in each category. LNI was defined as the combination of dNLR greater than 1.985 and LDH greater than 244 U/L, which separated patients in three different risk groups (Good: 0 factor; Intermediate: 1 factor; Poor: 2 factors). One and two factors were considered high risk and 0 factor was considered low risk. The relationship between clinical characteristics and the three parameters is shown in Table 3. The dNLR correlated significantly with HR status (*p* < 0.05).

**Table 3:** Associations Between Parameters and Clinicopathological Factors

Variables	dNLR			LDH			LNI		
	H	L	P	H	L	P	H LNI(1) + (2)	L	***P***
**Age (years)**									
< 60	12	31		17	26		23	20	
≥ 60	3	5	0.585	4	4	0.581	4	4	0.856
**ECOG PS**									
0	6	24		11	19		13	17	
≥ 1	9	12	0.078	10	11	0.434	14	7	0.1
**Menstrual status**									
Menopause	10	29		15	24		18	21	
Non-menopause	5	7	0.287	6	6	0.478	9	3	0.08
**CTCAE grades**									
≤ 2	12	26		18	20		23	15	
> 2	3	10	0.561	3	10	0.125	4	9	0.064
**Treatment lines**									
< 2	10	22		13	19		17	15	
≥ 2	5	14	0.708	8	11	0.917	10	9	0.973
**Brain metastases**									
No	14	27		15	26		21	20	
Yes	1	9	0.133	6	4	0.177	6	4	0.618
**Liver metastases**									
No	8	16		9	15		12	12	
Yes	7	20	0.562	12	15	0.615	15	12	0.692
**Anti-HER2 treatment**									
Yes	13	28		18	23		23	18	
No	2	8	0.466	3	7	0.423	4	6	0.360
**BMC status**									
De novo	0	5		2	3		2	3	
Recurrent	15	31	0.129	19	27	0.955	25	21	0.542
**HER2 status**									
HER2(IHC2+)FISH(+)	3	3		3	3		3	3	
HER2(IHC3+)	12	33	0.239	18	27	0.64	24	21	0.878
**HR status**									
Negative	5	24		11	18		12	17	
Positive	10	12	**0.029**	10	12	0.589	15	7	0.058
**Number of transfers**									
≤ 2	2	7		3	6		4	5	
> 2	13	29	0.602	18	24	0.598	23	19	0.574
**Prior surgery**									
No	0	5		2	3		2	3	
Yes	15	31	0.129	19	27	0.955	25	21	0.542

### PFS

**Fig. 1:**
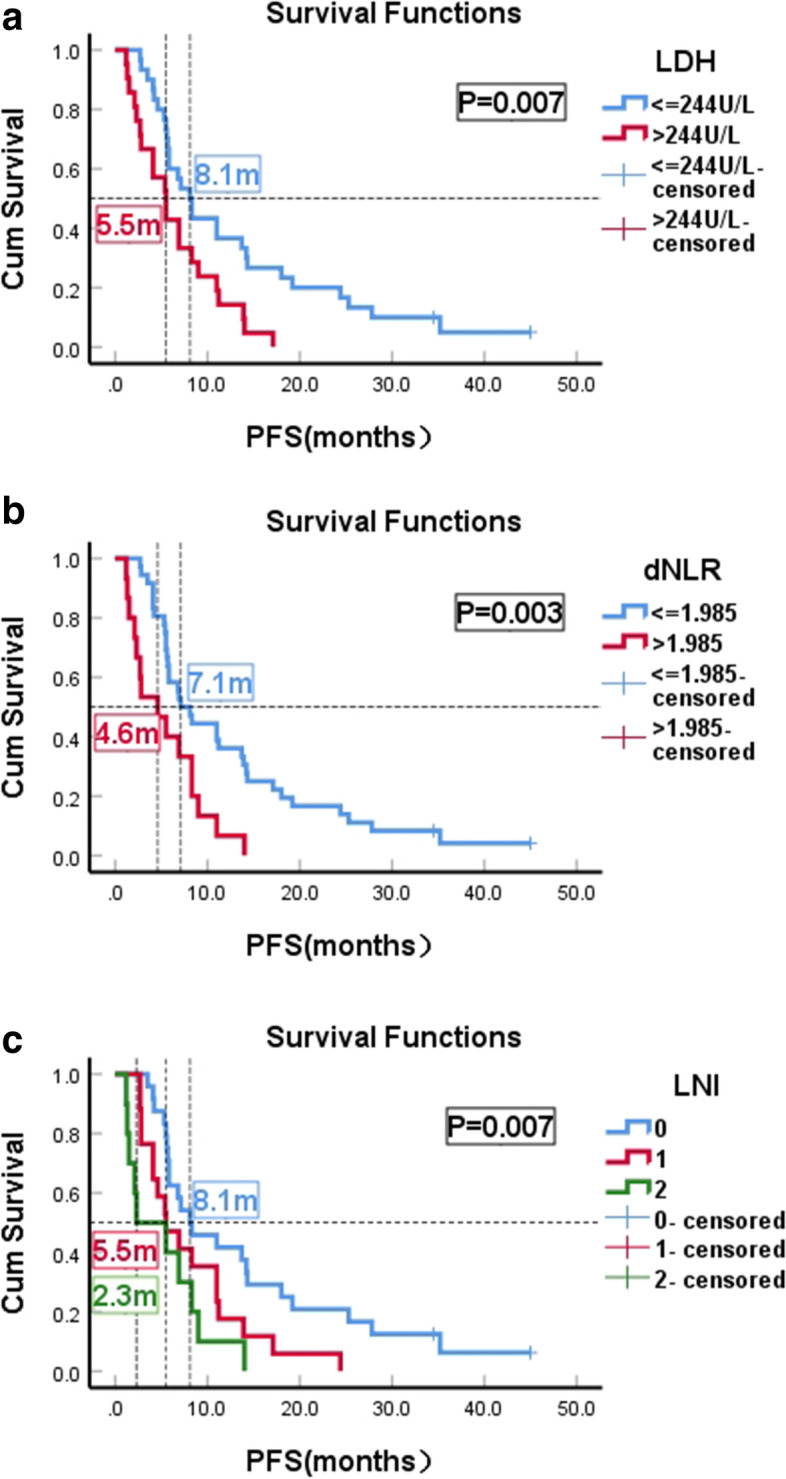
Kaplan-Meier PFS curves of HER2-Positive Advanced Breast Cancer Patients With T-DM1 Treatment. **a** Patients stratified according baseline LDH. **b** Patients stratified according baseline dNLR. **c** Patients stratified according LNI

When a baseline LDH value of 244 U/L was used as the cut-off, patients with LDH ≤ 244 U/L (*n* = 30; 58.8%;) had a significantly longer median PFS of 8.1 months (95% CI: 6.1–10.1) compared to patients with LDH>244 U/L (median PFS of 5.5 months, 95% CI: 3.4–7.6; *P* = 0.007). (Fig. 1a). Patients with baseline dNLR≤1.985 (*n* = 36; 70.6%) had a median PFS of 7.1 months (95% CI: 4.9–9.3) while patients with dNLR>1.985 (*n* = 15; 29.4%) had a median PFS of 4.6 months (95% CI: 1.1–8.1) (*P* = 0.003) (Fig. 1b). Among the 51 patients, the median PFS of LNI(0) (*n* = 24; 47%) LNI(1) (*n* = 17; 33%) and LNI(2) (*n* = 10; 20%) were 8.1 months(3.1–13.1 m) and 5.5 months(2.4–8.6 m) and 2.3 months(0–7.6 m), respectively, *P* = 0.007(Fig. 1c).

### OS

**Fig. 2:**
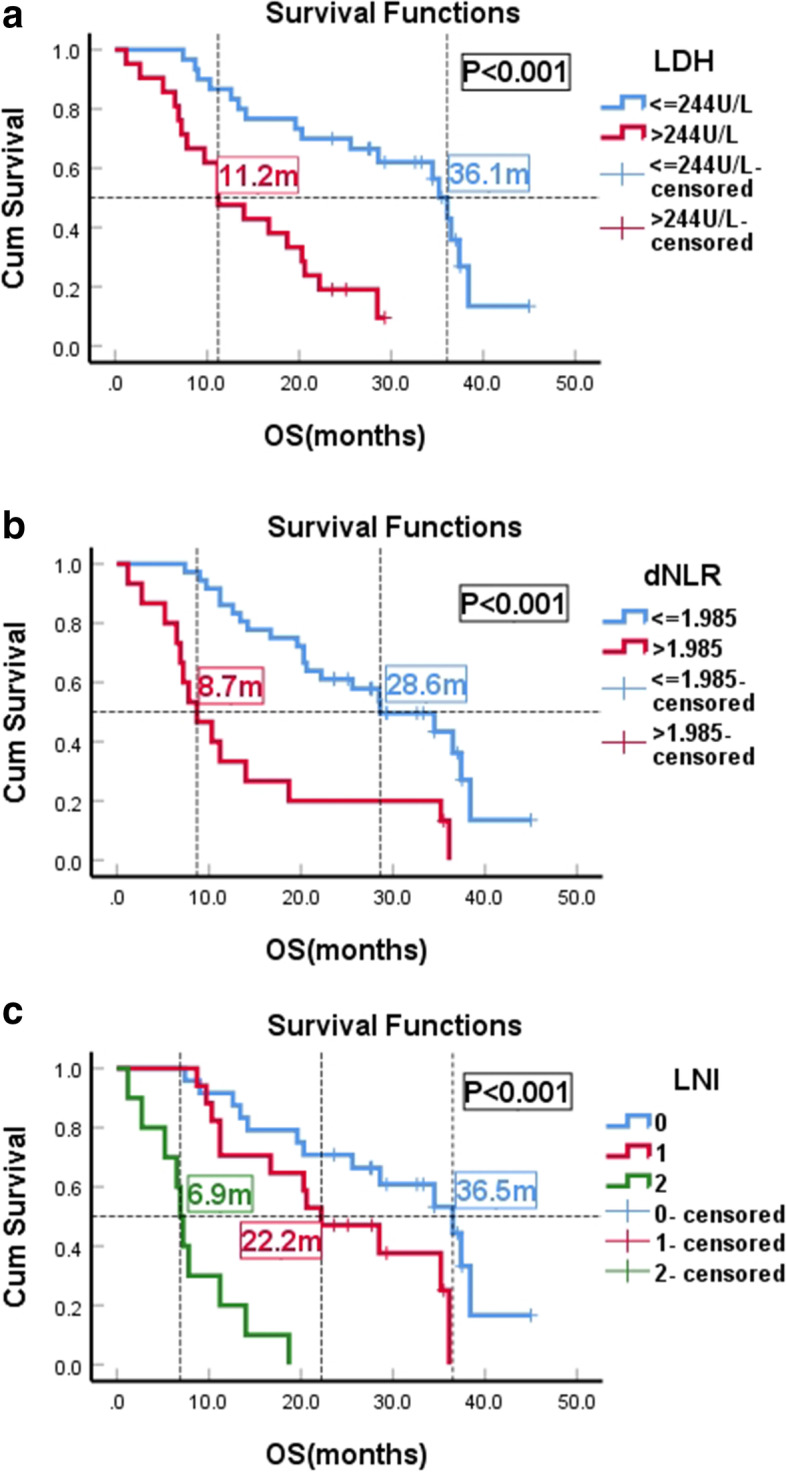
Kaplan-Meier OS curves of HER2-Positive Advanced Breast Cancer Patients With T-DM1 Treatment. 2a Patients stratified according baseline LDH. 2b Patients stratified according baseline dNLR. 2c Patients stratified according LNI

Median OS had significant difference between patients with baseline LDH>244 U/L and LDH ≤ 244 U/L(P<0.001) (Fig. 2a). Median OS was 8.7 months (95% CI: 4.8–12.6) for patients with baseline dNLR>1.985 compared with 28.6 months for patients with dNLR ≤1.985 (95% CI: 17.8–39.4) (*P* < 0.001) (Fig. 2b). The median OS of LNI(0) (*n* = 24; 47.1%) LNI(1) (*n* = 17; 33.3%)and LNI(2) (*n* = 10; 19.6%) were 36.5 months(12.7–31.7 m) and 22.2 months(13.0–31.4 m) and 6.9 months(5.8–8.0 m), respectively, (P<0.001) (Fig. 2c). There were significant differences among different layers.

### LDH and dNLR changing trend

**Fig. 3:**
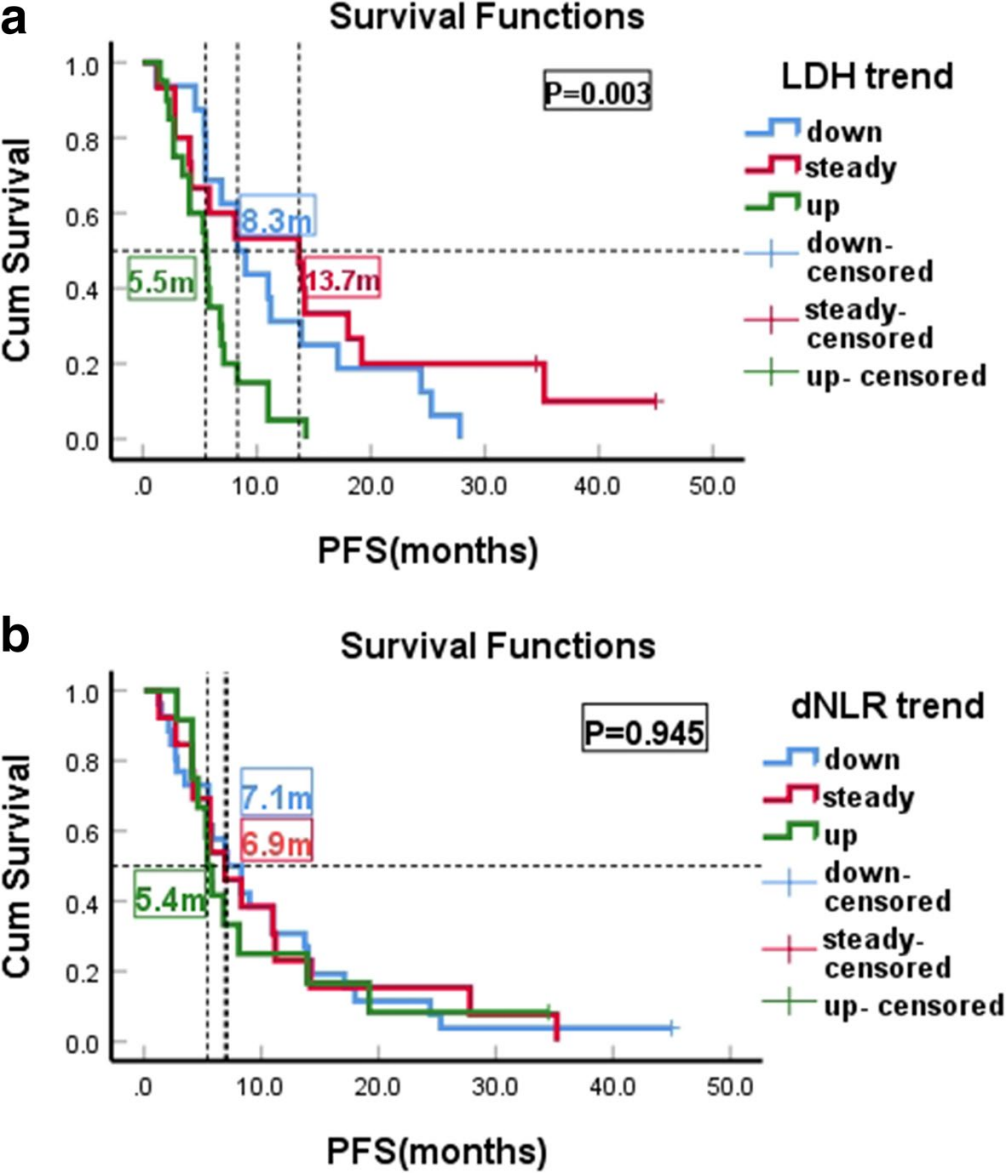
Kaplan-Meier PFS curves of HER2-Positive Advanced Breast Cancer Patients With T-DM1 Treatment. **a** Patients stratified according LDH changing trend. **b** Patients stratified according dNLR changing trend

**Fig. 4:**
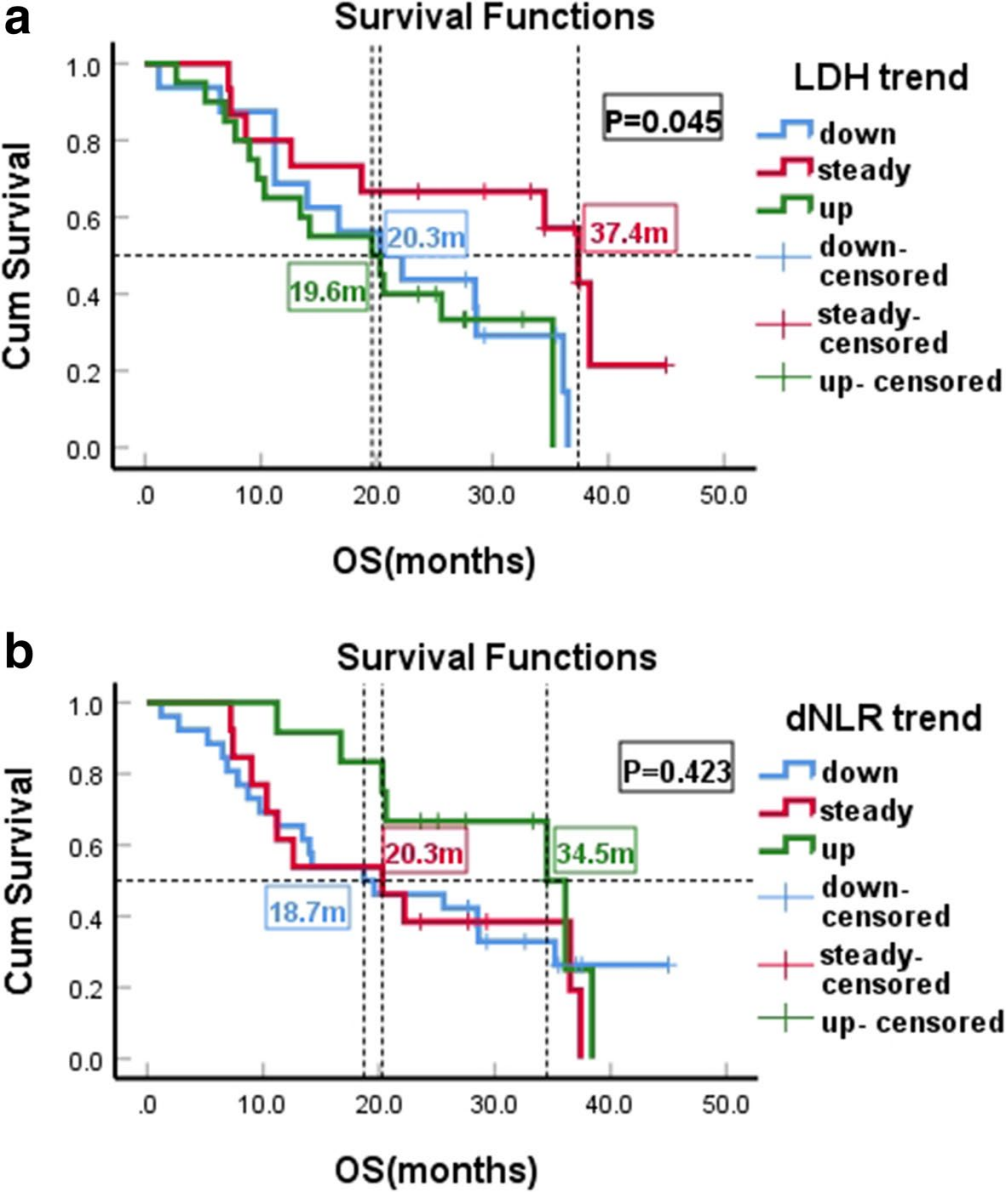
Kaplan-Meier OS curves of HER2-Positive Advanced Breast Cancer Patients With T-DM1 Treatment. **a** Patients stratified according LDH changing trend. **b** Patients stratified according dNLR changing trend

Among the LDH changes, 39.2% was uptrend, 29.4% steady and 31.4% downtrend. Median PFS was 5.5 months (95% CI: 4.8–6.2) (*P* = 0.003) for patients with LDH uptrend compared with 8.3 months for patients with downtrend (95% CI: 5.6–11.0) (*P* = 0.003), and 13.7 months for patients with LDH steady trend (95% CI: 3.3–24.1) (*P* = 0.003) **(**Fig. 3a**)**. However, no significant difference was observed between LDH downtrend and steady trend(*P* = 0.348). Elevated LDH level showed shorter median OS (19.6 months,95% CI: 6.23–33.0) (*P* = 0.045) **(**Fig. 4a**)**,but there was no significant differences between downtrend and uptrend (*P* = 0.499). In addition, the changing trends of dNLR had no significant effect on the PFS and OS of patients with T-DM1 treatment **(**Fig. 3 b, Fig. 4 b**)**.

### Univariate and multivariate analyses

Univariate and multivariate analyses of PFS and OS were performed by COX regression model, and considering factors included age, ECOG, thrombocytopenia CTCAE grades, menstrual status, treatment lines, HR status, HER2 status, number of metastatic sites, metastatic sites, treatment lines, anti-HER2 treatment, prior surgery, BMC status, pretreatment LDH level, pretreatment dNLR level, pretreatment LNI value, LDH and dNLR changing trend. In univariate analysis, we found that LDH > 244 U/L, dNLR> 1.985, LNI(1) and LNI(2) before T-DM1 treatment were associated with shorter PFS (HR, 2.238(1.217–4.116), *P* = 0.01; 2.549(1.330–4.888), *P* = 0.005; 2.260(1.130–4.522), *P* = 0.021; 3.193(1.438–7.091), *P* = 0.004) (Table 4), and OS (HR, 4.368(2.010–9.493), *P* < 0.001; 3.756(1.843–7.657), *P* < 0.001; 2.498(1.069–5.836), *P* = 0.035; 16.209(5.837–45.011), *P* < 0.001) (Table 5). Her-2(IHC3+), LDH downtrend and steady trend were associated with longer PFS (HR, 0.403(0.165–0.984), *P* = 0.046; 0.425(0.212–0.855), *P* = 0.016; 0.295(0.135–0.647), *P* = 0.002) (Table 4) and OS (HR, 0.172(0.063–0.470), *P* = 0.001;0.863(0.399–1.868), *P* = 0.709; 0.303(0.110–0.840), *P* = 0.022) (Table 5). Besides, ECOG PS > 1 was associated with shorter OS (HR, 2.587(1.302–5.140), *P* = 0.007) (Table 5).

**Table 4:** Univariate/Multivariate Analysis of PFS in HER2-Positive Advanced Breast Cancer Patients With T-DM1 Treatment

Variables	Univariate		Multivariate	
	HR(95% CI)	P	HR(95% CI)	P
**LDH**	2.238(1.217–4.116)	**0.01**	2.807(1.317–5.982)	**0.008**
**dNLR**	2.549(1.330–4.888)	**0.005**	1.668(0.809–3.439)	0.166
**LNI**				
**LNI(1)**	2.260(1.130–4.522)	**0.021**		
**LNI(2)**	3.193(1.438–7.091)	**0.004**		
**Age**	0.994(0.957–1.033)	0.775		
**ECOG PS**	1.085(0.611–1.927)	0.781		
**Menstrual status**	0.985(0.502–1.935)	0.966		
**Thrombocytopenia CTCAE grades**	1.395(0.732–2.658)	0.312		
**Treatment lines**	1.136(0.624–2.070)	0.676		
**Liver metastases**	1.206(0.686–2.122)	0.515		
**Brain metastasis**	1.678(0.827–3.406)	0.152		
**HER2 status**	0.403(0.165–0.984)	**0.046**		
**HR status**	1.490(0.843–2.634)	0.170		
**Number of metastatic sites**	0.861(0.360–2.059)	0.737		
**Prior surgery**	0.752(0.292–1.933)	0.554		
**Trastuzumab plus pertuzumab**	0.816(0.415–1.606)	0.556		
**Anti-HER2 treatment**	0.940(0.467–1.890)	0.816		
**BMC status**	0.752(0.292–1.933)	0.554		
**LDH changing trend**				
**Down**	0.425(0.212–0.855)	**0.016**	0.259(0.117–0.572)	**0.001**
**Steady**	0.295(0.135–0.647)	**0.002**	0.268(0.118–0.609)	**0.002**
**dNLR changing trend**				
**Down**	0.893(0.437–1.825)	0.756		
**Steady**	0.889(0.396–1.999)	0.777		

(Annotation: LDH ≤ 244 U/L, dNLR≤1.985, LNI(0), Age < 60 year, ECOG PS < 1, Premenopauseas, Thrombocytopenia CTCAE grades ≤2, Treatment lines < 2、No brain metastasis, No hepatic metastases, Her2(IHC2 + , FISH+), HR(−), Metastatic sites ≤2, No prior surgery,No trastuzumab plus pertuzumab treatment, No anti-HER2 treatment, de novo BMC,LDH uptrend, dNLR uptrend as references)

**Table 5:** Univariate/Multivariate Analysis of OS in HER2-Positive Advanced Breast Cancer Patients With T-DM1 Treatment

Variables	Univariate		Multivariate	
	HR(95% CI)	***P***	HR(95% CI)	P
**LDH**	4.368(2.010–9.493)	**< 0.001**		
**dNLR**	3.756(1.843–7.657)	**< 0.001**		
**LNI**				
**LNI(1)**	2.498(1.069–5.836)	**0.035**	2.063(0.818–5.203)	0.125
**LNI(2)**	16.209(5.837–45.011)	**< 0.001**	13.354(4.570–39.025)	**< 0.001**
**Age**	1.373(0.591–3.186)	0.461		
**ECOG PS**	2.587(1.302–5.140)	**0.007**	3.471(1.593–7.562)	**0.002**
**Menstrual status**	0.820(0.379–1.774)	0.614		
**Thrombocytopenia CTCAE grades**	1.515(0.718–3.197)	0.275		
**Treatment lines**	1.062(0.524–2.152)	0.869		
**Liver metastases**	1.574(0.790–3.136)	0.197		
**Brain metastasis**	1.095(0.451–2.658)	0.841		
**HER2 status**	0.172(0.063–0.470)	**0.001**	0.166(0.051–0.542)	**0.003**
**HR status**	1.382(0.700–2.729)	0.352		
**Number of metastatic sites**	1.360(0.560–3.300)	0.497		
**Prior surgery**	0.761(0.263–2.202)	0.614		
**Trastuzumab plus pertuzumab**	0.812(0.378–1.744)	0.593		
**Anti-HER2 treatment**	0.765(0.315–1.857)	0.554		
**BMC status**	0.761(0.263–2.202)	0.614		
**LDH changing trend**				
**Down**	0.863(0.399–1.868)	0.709	0.539(0.234–1.240)	0.146
**Steady**	0.303(0.110–0.840)	**0.022**	0.235(0.079–0.701)	**0.009**
**dNLR changing trend**				
**Down**	1.139(0.523–2.480)	0.743		
**Steady**	0.615(0.256–1.477)	0.277		

(Annotation: LDH ≤ 244 U/L, dNLR≤1.985, LNI(0), Age < 60 year, ECOG PS < 1, Premenopauseas, Thrombocytopenia CTCAE grades ≤2, Treatment lines < 2、No brain metastasis, No hepatic metastases, Her2(IHC2 + , FISH+), HR(−), Metastatic sites ≤2, No prior surgery,No trastuzumab plus pertuzumab treatment, No anti-HER2 treatment, de novo BMC,LDH uptrend, dNLR uptrend as references)

In the multivariate analysis of PFS, LDH > 244 U/L indicated poor prognosis (HR,2.807(1.317–5.982), *P* = 0.008) (Table 4), while it had no significant effect on the prognosis of OS. LDH downtrend or steady trend were independently correlated with poor prognosis of PFS (HR, 0.259(0.117–0.572), *P* = 0.001; 0.268(0.118–0.609), *P* = 0.002) (Table 4) and OS (HR, 0.539(0.234–1.240), *P* = 0.146; 0.235(0.079–0.701), *P* = 0.009) (Table 5). We found that in the multivariate analysis of OS, LNI (2) and ECOG PS > 1 could be used as adverse prognostic indicators (HR, 13.354(4.570–39.025), *P* < 0.001; 3.471(1.593–7.562), *P* = 0.002) (Table 5), Her-2(IHC3+) and LDH steady trend might indicate good prognosis (HR, 0.166(0.051–0.542), *P* = 0.003; 0.235(0.079–0.701), *P* = 0.009) (Table 5).

## Discussion

In the present study, the median OS and PFS of the 51 patients treated with T-DM1 were 22.2 m and 6.9 m, respectively, which was roughly consistent with the reported PFS of patients treated with T-DM1 [2, 16, 17]. As this study enrolled patients who were treated with T-DM1 as second-line, third-line and fourth-line therapy, and some patients did not reach OS when data collected, the median OS was lower than EMILIA study reported, but it was basically consistent with TH3RES research [16].

This study showed that LDH > 244 U/L before treatment was associated with poor prognosis in patients with advanced breast cancer treated with T-DM1. The median PFS and OS were 5.5 m and 11.2 m respectively. These results were similar to those reported in other advanced breast cancer [3, 10]. Serum LDH concentration is a surrogate marker for the metabolic activity of cancer cells. Studies have confirmed that in the tumor immune microenvironment, high concentrations of LDH can facilitate glycolysis under hypoxic conditions to provide energy, and can inhibit CD8 + T lymphocytes and natural killing (NK), and can also promote tumor angiogenesis [5–7]. LDH was found to be associated with shorter survival when its level rised up to 2.5 × ULN [4]. Therefore, LDH levels were included in the TNM staging system for melanoma [18]. In lymphoma [11, 12], melanoma [19], lung cancer [20], penile cancer [21] and other solid tumors, increased LDH is shown to be an adverse factor for the survival of patients.

In recent years, there have been several studies on using routine blood parameters as potential tumor prognostic markers, including C-reactive protein, albumin, neutrophil count, lymphocyte count and other leukocyte count [22, 23]. Comprehensive prognostic scores, such as GPS/mGPS and dNLR may standardize and maximize the prognostic value of cancers [24, 25]. High neutrophil and low lymphocytes can provide an appropriate environment favorable for tumor growth and metastasis to promote cancer development [13, 14]. NLR levels were positively correlated with the concentration of bone marrow-derived suppressive cells in peripheral blood, but negatively correlated with interferon-γ in breast cancer, suggesting that high NLR may represent an immunosuppressive state in the tumor microenvironment [26]. In this study we found that the increase of dNLR was also an adverse factor affecting the survival of patients with advanced breast cancer. The survival analysis of PFS and OS suggested that patients with dNLR> 1.985 had worse median PFS and OS (4.6 m, 95%CI 1.1–8.1, *P* = 0.003; 8.7 m,95%CI 4.8–12.6,*P* < 0.001). This was also similar to the threshold selected by other studies. Meanwhile, in univariate and multivariate analysis of PFS and OS, dNLR> 1.985 was also considered to be a worse prognostic factor. The results of this study were also consistent with the results of another study on the prognosis of advanced breast cancer [10]. Besides, in the study of predicting the prognosis of early breast cancer patients, the increase of dNLR was also a bad prognostic factor [27, 28].

LNI based on LDH and dNLR divided patients with advanced breast cancer in our study into three different risk groups (Good: 0 factor; Intermediate: 1 factor; Poor: 2 factors). Significant differences were observed between PFS and OS among the three groups. Patients with high-scoring LNI (LNI2) were more likely to progress and with the shortest median PFS(2.3 m) and median OS(6.9 m). It suggested that LNI might predict the prognosis of patients with advanced breast cancer treated with T-DM1. The prognostic value of LDH and dNLR was also confirmed in another study of metastatic breast cancer [10]. LIPI, a combined indicator of dNLR and LDH, has been widely used to study the prognosis of immunotherapy, especially in the studies of non-small cell lung cancer [19, 29] and melanoma [18, 30]. However, it is the first time to study the prognosis of advanced breast cancer treated with T-DM1 with LDH and dNLR combined parameters. The prognostic value of combined indicators needs more prospective studies to further confirm.

The results also showed that the status of HER2 affects the survival rate of patients. In the univariate analysis of PFS and OS, patients with HER2(IHC3+) had a better prognosis, and in the multivariate analysis of OS, HER2(IHC3+) still showed a better prognosis under the influence of LNI, ECOG scores and LDH changing trend. These results may be caused by that T-DM1 could bind more tightly to the receptor of cancer cells when HER2 highly expressed, and better exert the cytotoxic effect of DM1.This result was consistent with EMILIA [2] and TH3RESA [15] Phase III studies. These researches also showed that the objective remission rate of T-DM1 treatment was higher in patients whose HER-2 was definitely positive in the central laboratory. Patients with higher HER-2 mRNA expression level had longer PFS [31].

Based on the statistical results, the changing trend of LDH significantly affected the outcome of T-DM1 treatment in patients with HER2-positive metastatic breast cancer. In the survival analysis, it was seen that patients with LDH uptrend after 3 weeks of T-DM1 treatment had shorter PFS and OS, and LDH uptrend also showed higher risk in both univariate and multivariate analyses. It might be related to the LDH involvement in regulating cellular metabolism and tissue damage [30]. Furthermore, high levels of serum LDH can affect tumor progression and metastasis, and it may lead to poor prognosis of various cancers [3, 32–35]. However, in the survival analysis of OS, there were no significant differences in downtrend and uptrend, which might be related to the small sample size or other factors such as treatment after disease progressed. We may need a larger sample size or longer follow-up to determine the significance of this indicator.

According to the current NCCN guideline, trastuzumab plus pertuzumab is the first-line regimen in HER2 positive metastatic breast cancer. While, in this study, only 23.5% of patients were treated with trastuzumab plus pertuzumab, which may be related to the domestic listing time of patuzumab in China, patients’ economic conditions, treatment options et al. Besides, patients treated with trastuzumab plus pertuzumab in this study showed no significant survival advantages due to the small sample size. In the future, we need a larger sample size to evaluate the effect of dual-target combination therapy for HER2-positive breast cancer [36].

Due to the short marketing time of T-DM1 in China, fewer patients were able to use T-DM1 from May 2016 to November 2018. Besides, we could only collect the data on the use of T-DM1 in a single center. Therefore, the clinical available data of T-DM1 in patients with HER2-positive advanced breast cancer is indeed limited. Besides, as a retrospective study, we still can’t completely avoid recall bias although we have collected patients’ information as much as possible. In addition to those listed in the article, other factors such as previous chemotherapy and endocrine therapy may also affect the treatment outcome of T-DM1. Although there are still deficiencies in the research, we still hope that this study could provide guidance value for future large-sample statistics and prospective studies.

## Conclusion

In summary, LDH > 244 U/L,dNLR> 1.985, LNI with higher score before T-DM1 treatment and LDH uptrend after T-DM1 treatment are related to the poor results of T-DM1 treatment for advanced breast cancer. However, the statistical sample size of this study is small, and it is a single-center statistical study. We need a larger sample size, multi-center and prospective study to verify the role of this new score in T-DM1 treatment and further determine the predictive value of LNI.

## Data Availability

The data that support the findings of this study are available from the corresponding author upon reasonable request.

## Abbreviations

HER2: Human Epidermal Growth Factor Receptor2
T-DM1: Trastuzumab emtansine
dNLR: derived neutrophil-to-lymphocyte ratio
LDH: Lactate dehydrogenase;
LNI: LDH combined with dNLR index
ULN: Upper limit of normal value
ROC: Receiver operating characteristic curve
MBCs: Metastatic breast cancers
PFS: Progression-free survival
OS: Overall survival
HRs: Hazard ratios
CI: Confidence intervals
CT: Computerized tomography
RECIST 1.1: Response Evaluation Criteria 1.1 in Solid Tumors
CR: Complete response
PR: Partial response
SD: Stable disease
PD: Progressive disease
ECOG PS: Eastern Cooperative Oncology Group performance status
CTCAE: Common Terminology Criteria for Adverse Events
HR: Hormone Receptor
AUC: Area Under Curve
